# Aetiology and outcome of non-traumatic coma in African children: protocol for a systematic review and meta-analysis

**DOI:** 10.1186/s13643-021-01796-1

**Published:** 2021-10-29

**Authors:** Stephen T. J. Ray, Charlotte Fuller, Alexandra Boubour, Laura J. Bonnett, David G. Lalloo, Karl B. Seydel, Michael J. Griffiths

**Affiliations:** 1 Institute of Infection, Veterinary & Ecological Sciences, University of Liverpool, Liverpool, UK; 2 Malawi-Liverpool Wellcome Trust Clinical Research Programme, P.O. Box 30096, Chichiri, Blantyre, Malawi; 3 Blantyre Malaria Project, Blantyre, Malawi; 4 Leeds Children’s Hospital, Leeds Teaching Hospitals Trust, Leeds, UK; 5 Department of Neurology, Columbia University Irving Medical Center, New York, NY USA; 6 Department of Biostatistics, University of Liverpool, Liverpool, UK; 7 Liverpool School of Tropical Medicine, Liverpool, UK; 8 College of Osteopathic Medicine, Michigan State University, East Lansing, MI USA; 9 Department of Neurology, Alder Hey Children’s NHS Trust, Liverpool, UK; 10 National Institute for Health Research Health Protection Research Unit in Emerging and Zoonotic Infections, Liverpool, UK

**Keywords:** Coma, Non-traumatic, Aetiology, Children, Africa

## Abstract

**Background:**

Non-traumatic coma is a common acute childhood presentation to healthcare facilities in Africa and is associated with high morbidity and mortality. Historically, the majority of cases were attributed to cerebral malaria (CM). With the recent drastic reduction in malaria incidence, non-malarial coma is becoming a larger proportion of cases and determining the aetiology is diagnostically challenging, particularly in resource-limited settings. The purpose of this study will be to evaluate the aetiology and prognosis of non-traumatic coma in African children.

**Methods:**

With no date restrictions, systematic searches of MEDLINE, Embase, and Scopus will identify prospective and retrospective studies (including randomised controlled trials, cluster randomised trials, cohort studies, cross-sectional, and case-control studies) recruiting children (1 month–16 years) with non-traumatic coma (defined by Blantyre Coma Score ≤ 2 or comparable alternative) from any African country. Disease-specific studies will be included if coma is associated and reported. The primary outcome is to determine the aetiology (infectious and non-infectious) of non-traumatic coma in African children, with pooled prevalence estimates of causes (e.g., malaria). Secondary outcomes are to determine overall estimates of morbidity and mortality of all-cause non-traumatic coma and disease-specific states of non-traumatic coma, where available. Random effects meta-analysis will summarise aetiology data and in-hospital and post-discharge mortality. Heterogeneity will be quantified with *τ*^2^, *I*^2^, and Cochran’s *Q* test.

**Discussion:**

This systematic review will provide a summary of the best available evidence on the aetiology and outcome of non-traumatic coma in African children.

**Systematic review registration:**

PROSPERO CRD42020141937

**Supplementary Information:**

The online version contains supplementary material available at 10.1186/s13643-021-01796-1.

## Background

Non-traumatic coma is a common acute childhood presentation to healthcare facilities throughout Africa. Infectious causes of deep coma (defined as Blantyre Coma Scale (BCS) ≤ 2) include cerebral malaria (CM), acute bacterial meningitis (ABM), viral encephalitides, and HIV-associated opportunistic pathogens, such as tuberculosis and cryptococcal disease [1–3]. Non-infectious causes of deep coma include metabolic abnormalities and toxins [1–3]. In malaria-endemic regions, CM is diagnosed in children with coma and peripheral malaria parasitaemia with no other identifiable cause [1]. Limited diagnostic resources in many African settings and the phenomenon of asymptomatic malaria parasitaemia have rendered the alternate diagnoses of childhood coma under-described, resulting in a poor epidemiological understanding of this clinical presentation [4, 5]. A study in Kenya revealed that less than half of children admitted with acute non-traumatic coma had an identified cause [6]. With the recent drastic reduction in malaria incidence, it is postulated that non-malarial coma represents a larger proportion of coma cases [6]. Non-traumatic coma in children is associated with high mortality (15–58%) and significant neurological sequelae (31–68%) [7–9]. The long-term effects of coma on cognition and educational achievement are less commonly described, and existing literature primarily focuses on CM [10, 11]. These effects, including neurological impairment in cognitive and motor function, play an important role in the socioeconomic development of low-resource countries [12].

There is a paucity of data on the aetiology of non-malarial coma in Africa [3]. Given the high burden of morbidity and mortality associated with this clinical presentation, an overview of the literature to date is vital to direct further research. Gwer et al. performed a literature review of fourteen single studies on childhood non-traumatic coma in Africa and Asia [9]. Gwer et al. is the only existing review investigating childhood non-traumatic coma in Africa, and it does not include a formal outcome analysis nor meta-analysis (on aetiology or outcome). Gwer et al. also focused on all-cause non-traumatic coma studies and did not explore severe coma subgroups in single disease and/or pathogen studies for outcome. Moreover, since the publication of Gwer et al., there have subsequently been a number of larger important studies using molecular diagnostics and other adjuvant diagnostic capacities, such as magnetic resonance imaging (MRI) and electroencephalogram (EEG). Non-traumatic coma outcomes in Africa are heterogenous between studies, and long-term follow-up is sparse. A systematic review and meta-analysis on aetiology and outcome of paediatric non-traumatic coma in Africa is needed; it will substantially enrich accurate estimates of both the proportions of individual causes of non-traumatic coma and their associated morbidity and mortality.

This is a systematic review and meta-analysis seeking to evaluate the aetiology and prognosis of non-traumatic coma in African children. Using an adapted population, intervention, comparator, outcome, and study designs (PICOS) framework, we will examine the best available evidence on the aetiology of acute non-traumatic coma in African children, including specific syndromic cohorts in comatose children to broaden the search of studies and maximise the evidence base [13]. Our adapted framework will use the population (children with non-traumatic coma), exposure (aetiology of infection and covariates, e.g., HIV status and malnutrition), outcome (morbidity and mortality), and study design framework.

## Methods

### Protocol development and registration

The present protocol has been registered within the PROSPERO database (registration number CRD42020141937) and is being reported in accordance with the reporting guidance provided in the Preferred Reporting Items for Systematic Reviews and Meta-Analyses Protocols (PRISMA-P) statement [14] (see checklist in Additional file 1). We will use elements of the Cochrane Handbook of Systematic Reviews to guide the conducting of the planned systematic review [15].

### Outcomes of interest

The primary outcome of this study is to determine the aetiology (infectious and non-infectious) of non-traumatic coma in African children, with pooled prevalence estimates of causes (e.g., malaria). The secondary outcomes are to determine the overall estimates of morbidity (characterised as neurological sequelae or disability (cognitive and/or motor) related to the illness) and mortality of all-cause non-traumatic coma and disease-specific states of non-traumatic coma, where available.

### Eligibility criteria

We will consider for inclusion prospective and retrospective studies (randomised controlled trials, cluster randomised trials, cohort studies, cross-sectional, and case-control studies) recruiting children (1 month–16 years) with coma (defined by Blantyre Coma Score ≤ 2 or comparable alternative) from any African country. Disease-specific studies (e.g., malaria) will be included given that coma is associated and reported. Case reports, case series, commentaries, and editorials will be excluded from the review, and we will exclude all publications that do not contain primary data. This rationale for such a broad geographical and aetiological eligibility is to ensure the most comprehensive investigation and analysis of non-traumatic coma (including disease-specific studies) in African children to date.

### Information sources and search strategy

We will search the MEDLINE, Embase, and Scopus databases with no date restrictions. A draft search strategy for MEDLINE is available in Additional file 2. Searches will be limited to English and French articles. We will search conference proceedings and databases of ongoing studies (including ClinicalTrials.gov, ISRCTN registry, and the WHO ICTRP portal) to identify studies not found in the databases listed above; grey literature will not be included. When appropriate, attempts to contact study authors for primary data will be made. Reference lists of eligible studies and relevant systematic reviews will be searched to identify additional studies to be considered for inclusion. All records identified from the search will be imported to EndNote X9.3.1 (Clarivate Analytics, Philadelphia, PA), a citation management programme, following which duplicates will be removed.

### Screening and selection criteria

Two authors (SR and CF) will independently screen the title and abstract of each publication, and disagreements will be resolved by consensus or third author (AB). We will review a reference for the full text if this reference includes (1) a mention of coma, children (1 month–16 years) and non-traumatic aetiology and (2) recruitment in an African setting for which it is possible to disaggregate a total number of children with non-traumatic coma and possible to extract aetiology or outcome data. Those articles which do not clearly meet the inclusion criteria will be excluded. The number of records screened and excluded will be recorded.

The full text of the identified studies will be individually assessed for eligibility against the predetermined criteria by two reviewers (two out of CF, SR, and AB for each article) in duplicate, with disagreements resolved by consensus or third reviewer. Reasons for exclusion of full-text articles will be documented. Articles will be stored and managed in EndNote X9.3.1 (Clarivate Analytics, Philadelphia, PA).

### Data extraction

We will use the Cochrane Effective Practice and Organisation of Care (EPOC) standard data collection form and adapt it for study characteristics and outcome data [17]. Two review authors will pilot the form on a randomly selected subset of 10% of included studies [15, 18]. Pilot testing of the forms will include a computation of the reviewers’ reliability. Reviewers will extract data independently and in duplicate from each eligible study. Attempts to contact authors will be made to obtain missing outcome data or other key study characteristics. The full data extraction form can be found in Additional file 3.

### Assessment of risk of bias

Three reviewers (CF, SR, and AB) will independently conduct a bias risk assessment at the outcome and study-level to assess the quality of included studies. The revised Cochrane Risk-of-Bias tool (RoB 2.0) will be used for assessing randomised controlled trials [16]. A modified Newcastle-Ottawa Scale (NOS), which incorporates the Critical Appraisal Skills Programme (CASP) checklist and Risk of Bias in Non-Randomized Studies of Interventions (ROBINS-I), will be used for all other study types (Additional file 4) [19, 20]. Risk of bias for each domain will be recorded as high (1), low (0), or unclear. Any disagreements will be resolved by consensus or discussed with a fourth reviewer (MG) if consensus is not achieved. The risk of bias for included studies will be described throughout data synthesis.

### Data synthesis

A narrative synthesis of the data will also be provided, including a summary and explanation of characteristics and key summary tables and findings of included studies per outcome. For aetiology analyses, we will include all studies regardless of coma definition, and we will include usual care and intervention arms of RCTs. Heterogeneity will be quantified with *τ*^2^, *I*^2^, and Cochran’s *Q* test [15]. Aetiology data will be summarised by random effects meta-analysis. Because of concerns about meta-analysis of proportions on very heterogeneous populations, we also plan a meta-analysis of outcome stratified by inclusion criteria (e.g., coma measure). Mortality will be presented as a simple proportion with exact binomial confidence intervals, and pooled mortality estimates will be calculated using generalised linear mixed models (a normal-binomial model). For interventional studies, the outcomes in the usual care arm of the study only will be included in these estimates. There will also be a random effects meta-analysis of in-hospital and post-discharge mortality. Exploratory meta-regression will be undertaken to explore heterogeneity by including covariates as fixed effects (e.g. proportion of patients infected with HIV and malnutrition) and testing for improved model fit by likelihood ratio testing of nested models [21]. A *p*-value of < 0.05 will be considered a statistically significantly improved fit. Summary estimates of 1-month mortality, where available, will be considered together and presented in the same way. A sensitivity analysis will be performed in a subgroup analysis of malaria only including studies using Artemisinin derivatives.

Pooled prevalence estimates of malaria, acute bacterial or viral meningitis, encephalitis, and bloodstream infection will be calculated using random effects meta-analysis as above.

All studies will be included in the primary analysis. To assess meta-biases, we will conduct Funnel plots (when approximately ten studies are included in the meta-analysis) with an Egger test on asymmetry at alpha level 0.1 [22]. The strength of the body of evidence will be assessed using the GRADE approach [23]. Overall quality will be rated as high (further research is unlikely to change our confidence in the estimate of the effect), medium (further research is likely to have an impact on our confidence in the estimate of the effect), or low (further research is very likely to have an impact on our confidence in the estimate of the effect). Subgroup analyses will include meta-analysis of severe coma (very low coma score on admission), less severe coma (higher coma score on admission), and disease-specific aetiologies (malaria, bacterial meningitis, viral meningitis or encephalitis, or blood stream infection). High-quality studies — those graded low risk of bias across all domains — will be defined for each study type. A sensitivity analysis will be performed only including studies classified as medium- and low-risk, and a separate sensitivity analysis will be performed at the outcome-level using only high-quality studies [15].

## Discussion

We anticipate potential challenges in obtaining paper copies of journal articles via library resources, due to the effects of the SARS-CoV-2 pandemic on library staffing. In the event of protocol amendments, a description of the change and rationale will be documented with the journal, with the date of amendment.

There are some practical issues and limitations of our review. At the study-level, it will not always be possible to confidently ascertain depth of coma in these children, as a valid coma scale (e.g. Glasgow Coma Scale [GCS], BCS) is not always used in studies. At the review-level, more papers will likely be set in research centres and university hospitals, and fewer papers will be set at rural and district hospitals and clinics. Due to the endemicity of infectious febrile coma aetiologies (e.g. CM, ABM), more papers will likely be set in sub-Saharan Africa than North Africa. Together, these may lead to urban and regional biases. Lastly, included studies are limited to English and French, which may exclude papers written in other languages spoken throughout the continent.

This systematic review and meta-analysis will provide a transparent review of the available evidence to provide a more accurate understanding of the causes and outcomes of non-traumatic coma in African children. We seek to publish our results in a peer-reviewed journal, attend conferences, and inform relevant parties in the field of our findings. We hope that this review will raise awareness of this common presentation in African settings. This review will highlight gaps in the literature and areas in which future research is required.

## Supplementary Information

**Additional file 1.** PRISMA-P Checklist

**Additional file 2.** Draft of Search Strategy

**Additional file 3.** Draft of data extraction form

**Additional file 4.** Modified Newcastle Ottawa Scale

## Data Availability

Not applicable

## Abbreviations

CM: Cerebral malaria
ABM: Acute bacterial meningitis
MRI: Magnetic resonance imaging
EEG: Electroencephalogram
PRISMA-P: Preferred Reporting Items for Systematic Reviews and Meta-Analyses Protocols
PICOS: Population, Intervention, Comparator, Outcome, and Study Designs
HIV: Human immunodeficiency virus
EPOC: Effective Practice and Organisation of Care
NOS: Newcastle-Ottawa Scale
CASP: Critical Appraisal Skills Programme
