# Supplementary material for: Aetiology and outcome of non-traumatic coma in African children: protocol for a systematic review and meta-analysis
Source: Syst Rev. 2021 Oct 29;10:282. doi: 10.1186/s13643-021-01796-1 (PMC8556005; doi:10.1186/s13643-021-01796-1)
Supplement: Supplementary file 1 — Additional file 1. PRISMA-P Checklist [file 13643_2021_1796_MOESM1_ESM.docx]

**Additional file 1.** PRISMA-P Checklist

| **Section/Topic** | **#** | **Checklist Item** | **Information reported plus line number** | | |
| --- | --- | --- | --- | --- | --- |
| Identification | 1a | Identify the report as a protocol of a systematic review | Yes | 1-2 |  |
| Update | 1b | If the protocol is for an update of a previous systematic review, identify as such | No | N/A |  |
| Registration | 2 | If registered, provide the name of the registry (e.g., PROSPERO) and registration number in the Abstract | Yes | 70 |  |
| Contact | 3a | Provide name, institutional affiliation, and e-mail address of all protocol authors; provide physical mailing address of corresponding author | Yes | 4-35 |  |
| Contributions | 3b | Describe contributions of protocol authors and identify the guarantor of the review | Yes | 305-410 |  |
| Amendments | 4 | If the protocol represents an amendment of a previously completed or published protocol, identify as such and list changes; otherwise, state plan for documenting important protocol amendments | No | N/A |  |
| Sources | 5a | Indicate sources of financial or other support for the review | Yes | 299-303 |  |
| Sponsor | 5b | Provide name for the review funder and/or sponsor | Yes | 299-303 |  |
| Role of Sponsor/ Funder | 5c | Describe roles of funder(s), sponsor(s), and/or institution(s), if any, in developing the protocol | Yes | 299-303 |  |
| Rationale | 6 | Describe the rationale for the review in the context of what is already known | Yes | 97-139 |  |
| Objectives | 7 | Provide an explicit statement of the question(s) the review will address with reference to participants, interventions, comparators, and outcomes (PICO) | Yes | 132-154 |  |
| Eligibility Criteria | 8 | Specify the study characteristics (e.g., PICO, study design, setting, time frame) and report characteristics (e.g., years considered, language, publication status) to be used as criteria for eligibility for the review | Yes | 156-165 |  |
| Information Sources | 9 | Describe all intended information sources (e.g., electronic databases, contact with study authors, trial registers, or other grey literature sources) with planned dates of coverage | Yes | 167-177 |  |
| Search Strategy | 10 | Present draft of search strategy to be used for at least one electronic database, including planned limits, such that it could be repeated | Yes | Addi-tonal file 2 |  |
| Data Management | 11a | Describe the mechanism(s) that will be used to manage records and data throughout the review | Yes | 175-177; 191-192 |  |
| Selection Process | 11b | State the process that will be used for selecting studies (e.g., two independent reviewers) through each phase of the review (e.g., screening, eligibility, and inclusion in meta-analysis) | Yes | 179-192 |  |
| Data Collection Process | 11c | Describe planned method of extracting data from reports (e.g., piloting forms, done independently, in duplicate), any processes for obtaining and confirming data from investigators | Yes | 194-201 |  |
| Data Items | 12 | List and define all variables for which data will be sought (e.g., PICO items, funding sources), any pre-planned data assumptions and simplifications | Yes | Additional file 3 |  |
| Outcomes and Prioritization | 13 | List and define all outcomes for which data will be sought, including prioritization of main and additional outcomes, with rationale | Yes | 149-154 |  |
| Risk of Bias in Individual Studies | 14 | Describe anticipated methods for assessing risk of bias of individual studies, including whether this will be done at the outcome or study level, or both; state how this information will be used in data synthesis | Yes | 203-249 |  |
| Synthesis | 15a | Describe criteria under which study data will be quantitatively synthesized | Yes | 214-232 |  |
|  | 15b | If data are appropriate for quantitative synthesis, describe planned summary measures, methods of handling data, and methods of combining data from studies, including any planned exploration of consistency (e.g., *I* ^2^, Kendall’s tau) | Yes | 214-235 |  |
|  | 15c | Describe any proposed additional analyses (e.g., sensitivity or subgroup analyses, meta-regression) | Yes | 225-232; 243-249 |  |
|  | 15d | If quantitative synthesis is not appropriate, describe the type of summary planned | No | N/A |  |
| Meta-bias(es) | 16 | Specify any planned assessment of meta-bias(es) (e.g., publication bias across studies, selective reporting within studies) | Yes | 237-239 |  |
| Confidence in Cumulative Evidence | 17 | Describe how the strength of the body of evidence will be assessed (e.g., GRADE) | Yes | 239-240 |  |
