# Supplementary material for: Aetiology and outcome of non-traumatic coma in African children: protocol for a systematic review and meta-analysis
Source: Syst Rev. 2021 Oct 29;10:282. doi: 10.1186/s13643-021-01796-1 (PMC8556005; doi:10.1186/s13643-021-01796-1)
Supplement: Supplementary file 2 — Additional file 2. Draft of Search Strategy [file 13643_2021_1796_MOESM2_ESM.docx]

**Additional file 2.** Draft of Search Strategy*

| **Number** | **Search terms** |
| --- | --- |
| 1 | Coma* OR consciousness OR unconscious OR “non-traumatic coma” OR “non traumatic coma” OR “nontraumatic coma” OR encephalopathy OR “febrile encephalopathy” NOT “head injury” |
| 2 | aetiology OR aetiologies OR etiology OR etiologies OR cause OR causes OR causality |
| 3 | Paediatric OR pediatric OR child* |
| 4 | Africa OR “sub Saharan Africa” OR “sub-Saharan Africa” OR Algeria OR Angola OR Benin OR Botswana OR “Burkina Faso” OR Burundi OR Cameroon OR “Cape Verde” OR “Central African Republic” OR Chad OR Comoros OR “Republic of the Congo” OR “Democratic Republic of the Congo” OR “Cote d’Ivoire" OR Djibouti OR Egypt OR “Equatorial Guinea” OR Eritrea OR Eswatini OR Ethiopia OR Gabon OR “The Gambia” OR Ghana OR Guinea OR “Guinea-Bissau" OR Kenya OR Lesotho OR Liberia OR Libya OR Madagascar OR Malawi OR Mali OR Mauritania OR Mauritius OR Morocco OR Mozambique OR Namibia OR Niger OR Nigeria OR Rwanda OR “Sao Tome and Principe” OR Senegal OR Seychelles OR “Sierra Leone” OR Somalia OR “South Africa” OR “South Sudan” OR Sudan OR Swaziland OR Tanzania OR Togo OR Tunisia OR Uganda OR Zambia OR Zimbabwe |
| 5 | #1 AND #2 AND #3 AND #4 |

*Search was limited to English and French; timeline with no date restrictions.
