# Supplementary material for: Aetiology and outcome of non-traumatic coma in African children: protocol for a systematic review and meta-analysis
Source: Syst Rev. 2021 Oct 29;10:282. doi: 10.1186/s13643-021-01796-1 (PMC8556005; doi:10.1186/s13643-021-01796-1)
Supplement: Supplementary file 3 — Additional file 3. Draft of data extraction form [file 13643_2021_1796_MOESM3_ESM.docx]

**Additional file 3:** Draft of data extraction form.

**Summary Data**

| Reviewer name |  |
| --- | --- |
| Date form completed |  |
| Responses changed following reviewer consensus (specify responses) |  |

**Trial Unique Identifier**

e.g., Smith1999a

|  |
| --- |

**Source**

| Year of publication |  |
| --- | --- |
| Journal |  |
| PUBMED ID |  |
| Institutional sponsor and/or data custodian |  |
| Corresponding author  (if different to above) |  |

**Methods (Bias)**

| Trial Registration ID |  |
| --- | --- |
| Study design |  |
| Total study duration |  |
| Sequence generation |  |
| Allocation sequence  concealment |  |
| Blinding |  |
| Completeness of outcome data |  |
| Selective reporting |  |
| Other concerns about bias |  |

**Participants**

| Total number |  | |
| --- | --- | --- |
| Country |  | |
| Setting |  | |
| Inclusion criteria |  | |
| Exclusion criteria |  | |
| Withdrawals/exclusions |  | |
| Diagnostic criteria of  coma |  | |
| Age |  | |
| Sex | Males | |
| Methods of recruitment |  | |
| Average length of fever |  | |
| Seizures |  | |
| Type of seizure | Tonic clonic |  |
|  | Focal |  |
| Lactate |  | |
| BCS (or equivalent coma score) on admission |  | |
| Retinopathy |  | |
| EEG |  | |
| Cerebral imaging |  | |
| HIV status | Proportion tested |  |
|  | Proportion unknown |  |
|  | Proportion HIV+ |  |
|  | CD4 count |  |
|  | Proportion exposed |  |
| HIV ART treatment | Proportion on ART |  |
| Nutrition status | Proportion with malnutrition |  |
|  | Proportion with SAM |  |
|  | Proportion with wasting |  |
|  | Proportion with stunting |  |
| Admission details | Delay to hospital presentation |  |
|  | Time from admission to diagnosis |  |
|  | Time from admission to discharge |  |
|  | Duration of coma prior to admission |  |
|  | Depth of coma prior to admission |  |
| Other comorbidities |  |  |
| Other subgroups reported |  |  |

**Interventions (for RCT studies)**

| Number of  intervention groups |  | | |
| --- | --- | --- | --- |
| Regimen | **Regimen 1** | **Regimen 2** | **Regimen 3** |
| Intervention e.g., drug type |  |  |  |
| Number randomised to group |  |  |  |
| Duration of treatment period |  |  |  |
| Timing |  |  |  |
| Delivery |  |  |  |
| Providers |  |  |  |
| Co-interventions |  |  |  |

**Outcomes**

**Aetiology**

| Details of microbiological methods | *Microbiological microscopy and culture* |  |
| --- | --- | --- |
|  | *Molecular (PCR)* |  |
|  | *Type of PCR (single or multiplex)* |  |
|  | *Other (e.g., metagenomic sequencing* |  |
| Details of antimicrobial resistance testing |  | |
| % of culture positive cases |  | |
| % of culture negative cases |  | |
| **1 Aetiology**  Syndrome proportion  (e.g., ABM % of total cohort) | Cerebral malaria |  |
|  | CM with co-infection |  |
|  | ABM |  |
|  | Encephalitis |  |
|  | Sepsis |  |
|  | Unknown encephalopathy |  |
|  | Non-infective encephalopathy |  |
|  | Other (group) |  |
|  | Specify other if listed (e.g., neurocysticercosis) |  |
|  | Other 1 |  |
|  | Other 2 |  |
|  | Other 3 |  |
| **2 Aetiology**  Pathogen proportion per syndrome (e.g., Strep Pneumoniae % of total ABM; for all syndromes - e.g., ABM, encephalitis, etc.) | **Cerebral Malaria** |  |
|  | CM with co-infection (list pathogens) |  |
|  | **ABM** |  |
|  | Strep Pneumoniae |  |
|  | Salmonella spp (include type if available) |  |
|  | N. Meningitidis |  |
|  | H. Influenzae |  |
|  | E. coli |  |
|  | Klebsiella |  |
|  | Acinetobacter |  |
|  | Other |  |
|  | **Encephalitis** |  |
|  | HSV1 |  |
|  | HSV2 |  |
|  | CMV |  |
|  | VZV |  |
|  | Enterovirus |  |
|  | Other |  |
|  | **Sepsis** |  |
|  | Staphylococcal aureus |  |
|  | N. Meningitidis |  |
|  | H. Influenzae |  |
|  | E. coli |  |
|  | Klebsiella |  |
|  | Acinetobacter |  |
|  | **Unknown Encephalopathy** |  |
|  | **Other (group)** |  |
|  | Specify other if listed e.g. neurocysticercosis |  |
|  | Other 1 |  |
|  | Other 2 |  |
|  | Other 3 |  |

**Clinical Outcome**

**Outcomes**

Notes:

For interventional studies, the outcomes in the usual care arm of the study only were included

| Outcome measures used | Neurological Sequelae |  |
| --- | --- | --- |
|  | Death |  |
|  | Other (e.g., neurocognitive assessments) |  |
|  | *Please specify:* | |
| Duration of follow-up |  | |

**Disability**

| **Outcome 1**  Disability proportion in entire cohort (e.g., 35% had neurological sequelae) |  |
| --- | --- |

| **Outcome 2**  Disability proportion in each syndrome (e.g., 35% had neurological sequelae in ABM) | Cerebral malaria |  |
| --- | --- | --- |
|  | CM with co-infection |  |
|  | ABM |  |
|  | Encephalitis |  |
|  | Sepsis |  |
|  | Unknown encephalopathy |  |
|  | Other (group) |  |
|  | Specify other if listed (e.g., neurocysticercosis) |  |
|  | Other 1 |  |
|  | Other 2 |  |
|  | Other 3 |  |

| **Outcome 3**  Disability proportion in each pathogen (e.g., 35% had neurological sequelae with Strep Pneumoniae) | **Cerebral Malaria** |  |
| --- | --- | --- |
|  | CM with co-infection (list pathogens) |  |
|  | **ABM** |  |
|  | Strep Pneumoniae |  |
|  | Salmonella spp (include type if available) |  |
|  | N. Meningitidis |  |
|  | H. Influenzae |  |
|  | E. coli |  |
|  | Klebsiella |  |
|  | Acinetobacter |  |
|  | Other |  |
|  | **Encephalitis** |  |
|  | HSV1 |  |
|  | HSV2 |  |
|  | CMV |  |
|  | VZV |  |
|  | Enterovirus |  |
|  | Other |  |
|  | **Sepsis** |  |
|  | N. Meningitidis |  |
|  | H. Influenzae |  |
|  | E. coli |  |
|  | Klebsiella |  |
|  | Acinetobacter |  |
|  | **Unknown Encephalopathy** |  |
|  | **Other (group)** |  |
|  | Specify other if listed (e.g., neurocysticercosis) |  |
|  | Other 1 |  |
|  | Other 2 |  |
|  | Other 3 |  |

| **Outcome 4**  Proportion of disability type per clinical diagnosis (e.g., % age ABM patients with deafness) | Cerebral malaria | *Motor*  *Cognitive/behavioural*  *Visual impairment*  *Hearing impairment*  *Epilepsy* |  |
| --- | --- | --- | --- |
|  | CM with co-infection | *Motor*  *Cognitive/behavioural*  *Visual impairment*  *Hearing impairment*  *Epilepsy* |  |
|  | ABM | *Motor*  *Cognitive/behavioural*  *Visual impairment*  *Hearing impairment*  *Epilepsy* |  |
|  | Encephalitis | *Motor*  *Cognitive/behavioural*  *Visual impairment*  *Hearing impairment*  *Epilepsy* |  |
|  | Sepsis | *Motor*  *Cognitive/behavioural*  *Visual impairment*  *Hearing impairment*  *Epilepsy* |  |
|  | Unknown encephalopathy | *Motor*  *Cognitive/behavioural*  *Visual impairment*  *Hearing impairment*  *Epilepsy* |  |
|  | Other (group) | *Motor*  *Cognitive/behavioural*  *Visual impairment*  *Hearing impairment*  *Epilepsy* |  |
|  | Specify other if listed (e.g., neurocysticercosis) |  |  |
|  | Other 1 |  |  |
|  | Other 2 |  |  |
|  | Other 3 |  |  |

**Death**

| **Outcome 1**  Death proportion in entire cohort (e.g., 35% died) |  |
| --- | --- |

| **Outcome 2**  Death proportion in each syndrome (e.g., 35% died in ABM) | Cerebral Malaria |  |
| --- | --- | --- |
|  | CM with co-infection |  |
|  | ABM |  |
|  | Encephalitis |  |
|  | Sepsis |  |
|  | Unknown Encephalopathy |  |
|  | Other (group) |  |
|  | Specify other if listed (e.g., neurocysticercosis) |  |
|  | Other 1 |  |
|  | Other 2 |  |
|  | Other 3 |  |

| **Outcome 3**  Death proportion in each pathogen (e.g., 35% of those with Strep Pneumoniae ABM died) | **Cerebral Malaria** |  |
| --- | --- | --- |
|  | CM with co-infection (list pathogens) |  |
|  | **ABM** |  |
|  | Strep Pneumonia |  |
|  | Salmonella spp (include type if available) |  |
|  | N. Meningitidis |  |
|  | H. Influenzae |  |
|  | E. coli |  |
|  | Klebsiella |  |
|  | Acinetobacter |  |
|  | Other |  |
|  | **Encephalitis** |  |
|  | HSV1 |  |
|  | HSV2 |  |
|  | CMV |  |
|  | VZV |  |
|  | Enterovirus |  |
|  | Other |  |
|  | **Sepsis** |  |
|  | Staphylococcal aureus |  |
|  | N. Meningitidis |  |
|  | H. Influenzae |  |
|  | E. coli |  |
|  | Klebsiella |  |
|  | Acinetobacter |  |
|  | **Unknown Encephalopathy** |  |
|  | **Other (group)** |  |
|  | Specify other if listed (e.g., neurocysticercosis) |  |
|  | Other 1 |  |
|  | Other 2 |  |
|  | Other 3 |  |

**Other Information**

| Comments on statistical methods |  |
| --- | --- |
| Have important populations been excluded from the study? |  |
| Key conclusions of study authors |  |
| Funding source |  |
