# Supplementary material for: Aetiology and outcome of non-traumatic coma in African children: protocol for a systematic review and meta-analysis
Source: Syst Rev. 2021 Oct 29;10:282. doi: 10.1186/s13643-021-01796-1 (PMC8556005; doi:10.1186/s13643-021-01796-1)
Supplement: Supplementary file 4 — Additional file 4. Modified Newcastle Ottawa Scale [file 13643_2021_1796_MOESM4_ESM.docx]

**Additional file 4:** Modified Newcastle Ottawa Scale

Note: A study can be awarded a maximum of one star for each numbered item within the Selection and Outcome categories. A maximum of two stars can be given for Comparability Selection.

**Representativeness of the exposed cohort (max 1)**

a) Truly representative of the average child in coma in the community*

b) Somewhat representative of the average child in coma in the community*

c) Selected group of users - e.g., nurses, volunteers

d) No description of the derivation of the cohort

**Selection of the non-exposed cohort (max 1)**

a) Drawn from the same community as the exposed cohort*

b) Drawn from a different source

c) No description of the derivation of the non-exposed cohort

**Ascertainment of exposure**

**Coma definition (max 1)**

a) Clear definition of coma using validated coma scale - e.g., BCS, GCS*

b) Clear definition of coma using non-validated coma scale - e.g., “severe coma defined as withdrawal to pain only”*

c) No clear definition of coma, not using a scale - e.g., “coma/altered consciousness” but no reference to how this was defined

**Aetiology (1) (max 1)**

a) Reproducible description of testing procedures using reference tests - e.g., PCR testing for pathogens*

b) Reproducible description of testing using non-reference tests - e.g., novel unvalidated serology testing

c) No or non-reproducible description of testing

**Aetiology (2) (max 1)**

a) Reproducible testing for pathogens, and attribution of the suspected or confirmed brain infection to the responsible pathogen, applied in a standard, reliable way for all participants*

b) Participants did NOT undergo the same panel or stepwise approach to testing, AND/OR clear unambiguous methods for defining causality of the pathogens were NOT reported or implied.

**Aetiology (3) (max 1)**

a) Denominator (participants with suspected or confirmed brain infection), and numerators (participants with identified pathogens) consistent and accurate, according to the methods and all reported frequencies*

b) There are inconsistencies or inaccuracies in the numerators or denominators.

**Comparability of cohorts on the basis of the design or analysis (max 3)**

a) Study reports +/- controls level of coma, etc.*

b) Study reports +/- controls for any additional physiologic laboratory markers/parameters (e.g., lactate, parasitaemia, BP, HR, etc.)*

c) Study reports +/- controls adjusted for age, expected development milestones, socio-economic status in relation to cognitive development post-illness*

d) No described adjustment for confounding factors

**Outcome of Interest (max 1)**

a) Independent blind assessment*

b) Record linkage*

c) Self-report

d) No description

**Adequacy of follow up (max 1)**

a) Complete follow up – all subjects accounted for*

b) Subjected lost to follow up unlikely to introduce bias – small number lost - >90%*

c) Follow up rate <90% and no description of those lost

d) No statement
